# A display and analysis platform for gut microbiomes of minority people and phenotypic data in China

**DOI:** 10.1038/s41598-023-36754-5

**Published:** 2023-08-30

**Authors:** Jun Li, Chunxue Wei, Ting Zhou, Chunfen Mo, Guanjun Wang, Feng He, Pengyu Wang, Ling Qin, Fujun Peng

**Affiliations:** 1 Department of Gastroenterology, The First Affiliated Hospital of Chengdu Medical College, 278# Bao Guang Road, Xindu District, Chengdu, 610000 Sichuan People’s Republic of China; 2 Department of Gastroenterology, The Sixth People’s Hospital of Chengdu, Chengdu, Sichuan China; 3 Department of Immunology, School of Basic Medical Sciences, Chengdu Medical College, Chengdu, Sichuan China; 4 College of Pharmacy, Chengdu Medical College, Chengdu, Sichuan China; 5 Institute of Basic Medicine, Weifang Medical University, 7166# Baotong West Road, Weifang, 261053 Shandong People’s Republic of China

**Keywords:** Microbiology, Diseases, Gastroenterology

## Abstract

The minority people panmicrobial community database (MPPCD website: http://mppmcdb.cloudna.cn/) is the first microbe-disease association database of Chinese ethnic minorities. To research the relationships between intestinal microbes and diseases/health in the ethnic minorities, we collected the microbes of the Han people for comparison. Based on the data, such as age, among the different ethnic groups of the different regions of Sichuan Province, MPPCD not only provided the gut microbial composition but also presented the relative abundance value at the phylum, class, order, family and genus levels in different groups. In addition, differential analysis was performed in different microbes in the two different groups, which contributed to exploring the difference in intestinal microbe structures between the two groups. Meanwhile, a series of related factors, including age, sex, body mass index, ethnicity, physical condition, and living altitude, were included in the MPPCD, with special focus on living altitude. To date, this is the first intestinal microbe database to introduce altitude features. In conclusion, we hope that MPPCD will serve as a fundamental research support for the relationship between human gut microbes and host health and disease, especially in ethnic minorities.

## Introduction

In 2001, the Human Genome Project (HGP) was completed as a result of the efforts of six countries. The project highlights the importance of understanding the synergistic activities between humans and microbes^1–3^. Subsequently, many biologists, especially microbiologists, immunologists, and medical scientists, began to explore the relationship between human diseases and the gut microbiota^4,5^. Meanwhile, large research projects, such as the Human Microbiome Project (HMP) and MetaHIT, have provided new ideas for microbiome research and enhanced biologists’ understanding of host-microbe interactions, especially the human gut microbiome with clinical functions^6,7^.

In normal conditions, gut microbes maintain a symbiotic relationship with hosts to help humans to digest and absorb nutrients, to provide energy, to produce important metabolites and bioactive components, to affect the immune system development, to regulate immune mediators, to maintain a normal intestinal barrier, and to prevent the infestation of pathogens^8,9^. Therefore, the gut microbe genomes are also regarded as “our second genome”. Many countries and organizations have established gut microbiome projects, including the GUT Project in the United States, the Chinese Academy of Sciences Microbiome Project, and the Canadian Microbiome Project, aimed at better understanding gut ecosystems and their functions^10^. Dysbiosis in the microbiota can lead to a series of diseases, including asthma, polycystic ovary syndrome, obesity, type 2 diabetes, and hypertension^4,11–15^. Currently, there are several public platforms that relate the microbiome and these diseases, such as gcMeta^16^, MANTA^17^, HMDAD^18^, and HPMCD^19^.

The human gut is dominated by 150–170 species of bacteria that perform protective, metabolic and structural functions^10^. However, a large number of studies have demonstrated that the species and relative abundance of gut microbes are associated with a series of factors, including diet, age, sample type (health or disease), gender, ethnicity, and weight^10,20–22^. Numerous studies have found that differing dietary patterns could affect microbe compositions^23–25^. One case—control study showed that *Enterobacteriaceae* was obviously reduced in vegans compared to omnivorous control populations^26^. Experimental mice fed a high-fat diet (40–80% total caloric intake) exhibit decreased *Bacteroidetes* at the phylum level and increased *Firmicutes*^24^. The analyses of faecal samples in the four European study groups indicated age-related structural differences in the bacterial communities^27^. *Bacteroides* presented lower levels in elderly subjects than in younger subjects from Italy^28^. In addition, the diversity of gut microbes is caused by ethnicity and sex^27,29, 30^.

There are currently some common platforms for microbiological disease-related databases. For example, HMDAD sorts out the relationship between microbes and diseases from previously published microbiome studies, and studies how they change by integrating disease genes, symptoms, chemical fragments and drugs^31^. MicroPhenoDB describes the relationship between microbial core genes and disease phenotypes by integrating microbial disease association data^32^. HPMCD provides a metagenomic resource that enables analysis of the gut microbiome and can search for health or disease states associated with the presence of the microbiome^33^. However, these databases only emphasize the relationship between gut microbes and hosts or briefly classify the subjects who are an important effective factor. In this study, we focused on the associations among the populations in ethnic minorities of Sichuan Province of China and gut microbes. First, we recruited persons from different regions in Sichuan Province, gathered their faeces, and acquired their clinical information, such as age and weight. Then, genomic DNA of each individual was extracted, the 16S rRNA of which was sequenced. Furthermore, we performed a series of processing for these datasets from gut microbial communities. Finally, these results and analysis were presented in the MPPCD database. Previously, we have completed the evolutionary analysis of intestinal microbes of Tibetan, laying a foundation for the establishment of MPPCD.

## Results

### Clinical characteristics of all the samples

**Figure 1:**
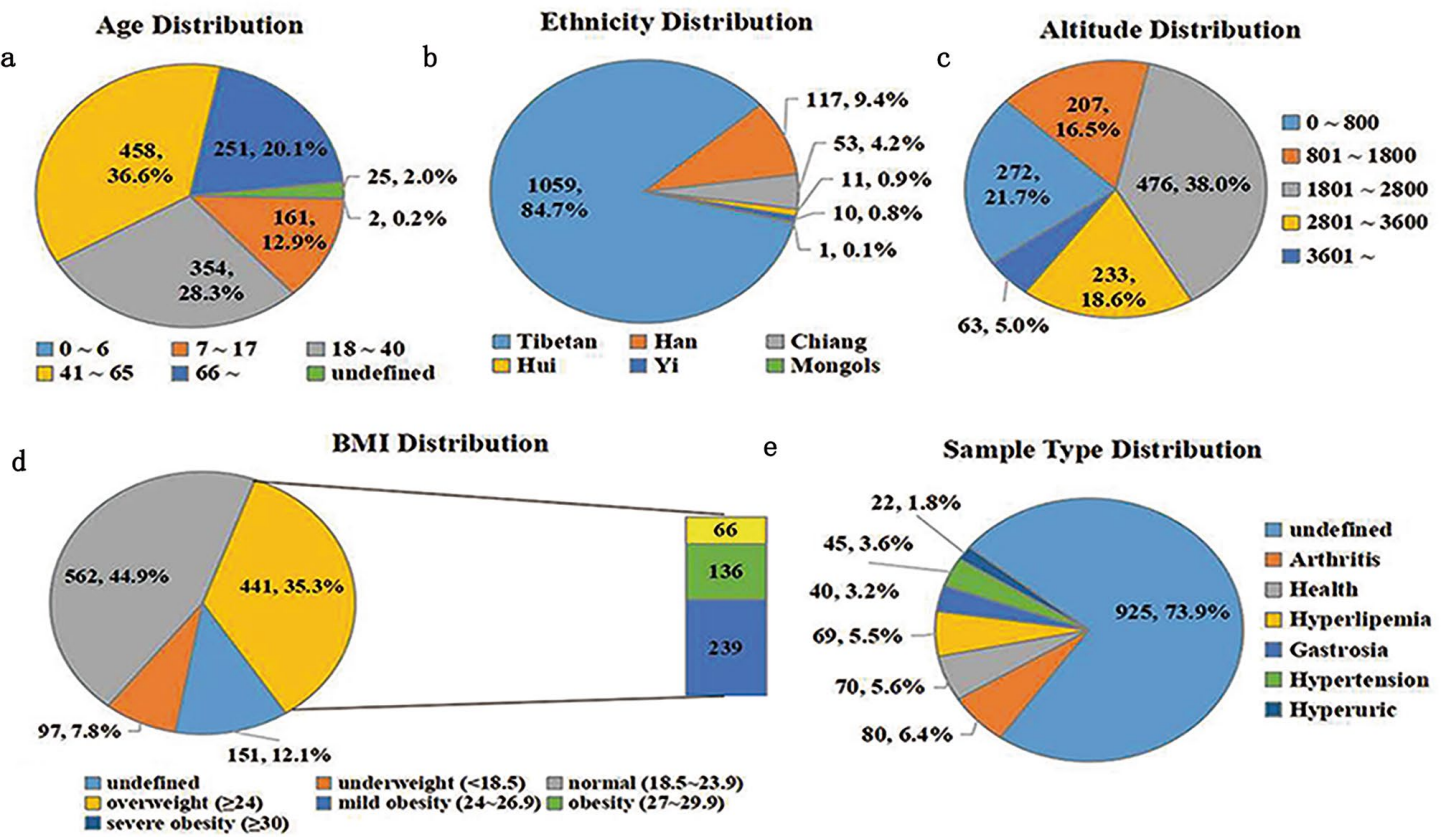
Clinical characteristics of all samples. (**a**) Age distribution analysis of all samples. (**b**) Ethnicity distribution analysis of all the samples. (**c**) Analysis of living altitude distribution of all samples. (**d**) BMI distribution analysis of all samples. (**e**) Health status distribution analysis of all samples.

All the individuals belong to different ethnic groups and live in different areas of Sichuan province, ranging from 500 to 4001 m above sea level. The detailed and statistical results are shown in Fig. 1. Regarding age, we performed statistical distribution according to the < Chinese Modern Age Division Standard > and found that the populations of middle age (41–65 years old), old age (≥ 66 years old) and young age (18–40 years old) were the top three groups (Fig. 1a). In the different ethnic groups, the ordering was Tibetan, Han and Chiang Chinese (Fig. 1b). Most people live between 800 and 3600 m above sea level, and a few people live less than 800 m or more than 3600 m above sea level (Fig. 1c). The BMI ordering was 562 normal, 441 overweight, 151 undefined, and 97 underweight. Meanwhile, the overweight populations were divided into mild obesity, obesity, and severe obesity according to World Health Organization Standards (Fig. 1d). Arthritis, hyperlipidemia, hypertension, gastrosia, and hyperuric were the most common diseases among the participants (Fig. 1e).

### Disease association analysis

**Figure 2:**
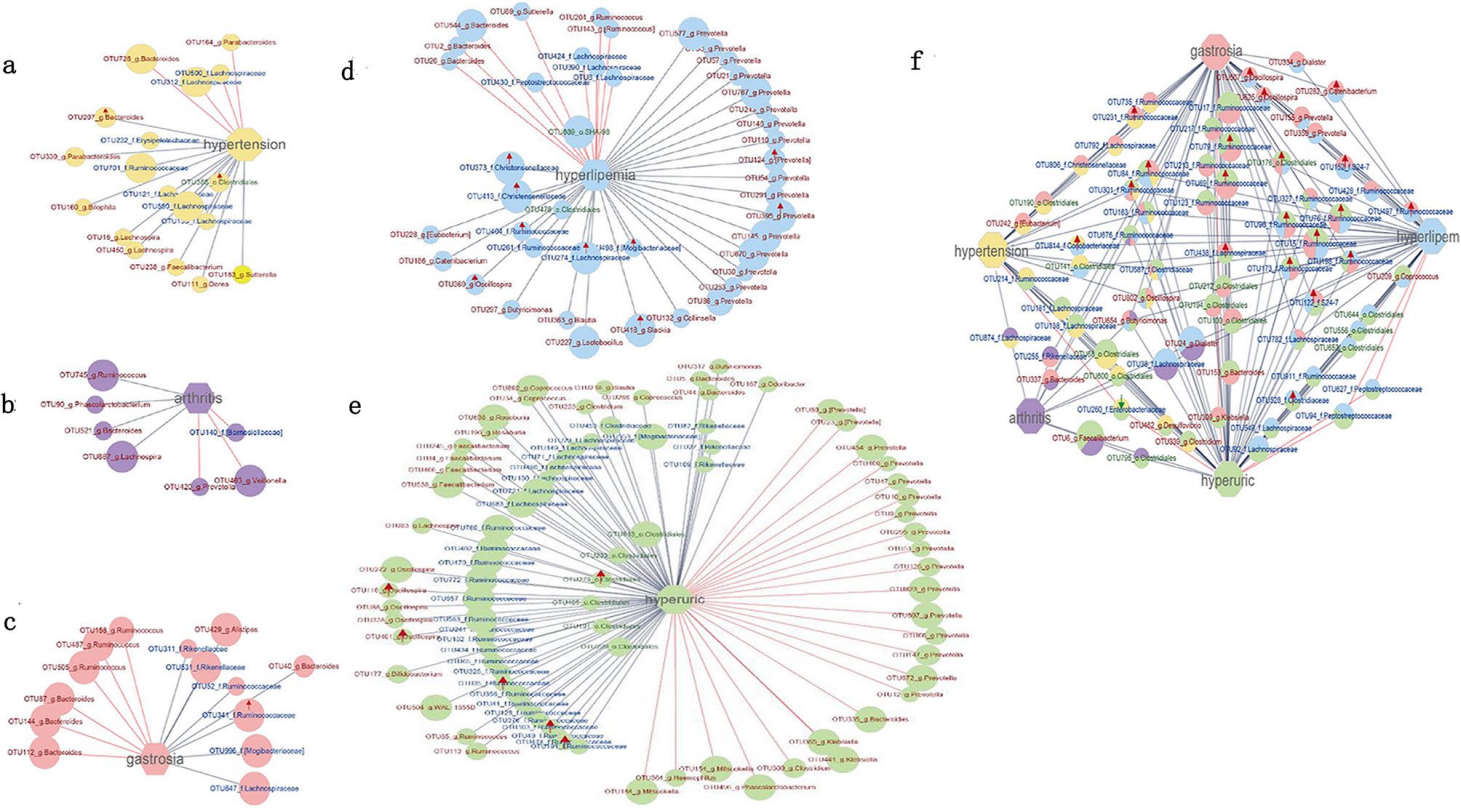
The relationship between living altitude, intestinal microbes and disease. (**a**) The relationship between hypertension and intestinal microbes and altitude. (**b**) The relationship between arthritis and intestinal microbes and altitude. (**c**) The relationship between chronic gastritis and intestinal microbes and altitude. (**d**) The relationship between hyperlipemia and intestinal microbes and altitude. (**e**) The relationship between hyperuria and intestinal microbes and altitude. (**f**) The relationship between these five diseases and the intestinal microbes and the altitude.

In order to further study the relationship between intestinal microbes and diseases, we selected 5 diseases with high prevalence in Tibetan population, including rheumatoid arthritis, hyperlipidemia, hypertension, chronic gastritis, and hyperuricemia. Based on the effective data of each sample: QIIME software (Version 1.9.1) was used to cluster OTUs. During the clustering process, the valid data of all samples were combined and the sequence was classified into an OTU with 97% similarity. OTU with significant difference in abundance between each disease and the normal group was found, and the network graph was constructed (Fig. 2a–e). In addition, OTU with differences in more than one disease were screened out to construct a network diagram (Fig. 2f). Correlation analysis is conducted between OTU and altitude. The red upward arrow represents a significant positive correlation between OTU and altitude, and the green downward arrow represents a significant negative correlation between OTU and altitude. The results show that that there was a significant OTU difference in the abundance of Bacteroides between the hypertensive group and the normal group, and it was positively correlated with altitude (Fig. 2a). OTU of *Ruminococcaceae* abundance was significantly different between gastritis group and normal group, and was positively correlated with altitude (Fig. 2c). The abundance of OTU such as *Ruminococcaceae* and *Oscillospira* was significantly different between the hyperlipemia group, the hyperuric group and the normal group, and was positively correlated with altitude (Fig. 2d–e). At the same time, *Ruminococcaceae* and *Oscillospira* show significant differences OTUs in a variety of diseases, and are positively correlated with altitude (Fig. 2f).

### The influence of external factors on intestinal microbes

**Figure 3:**
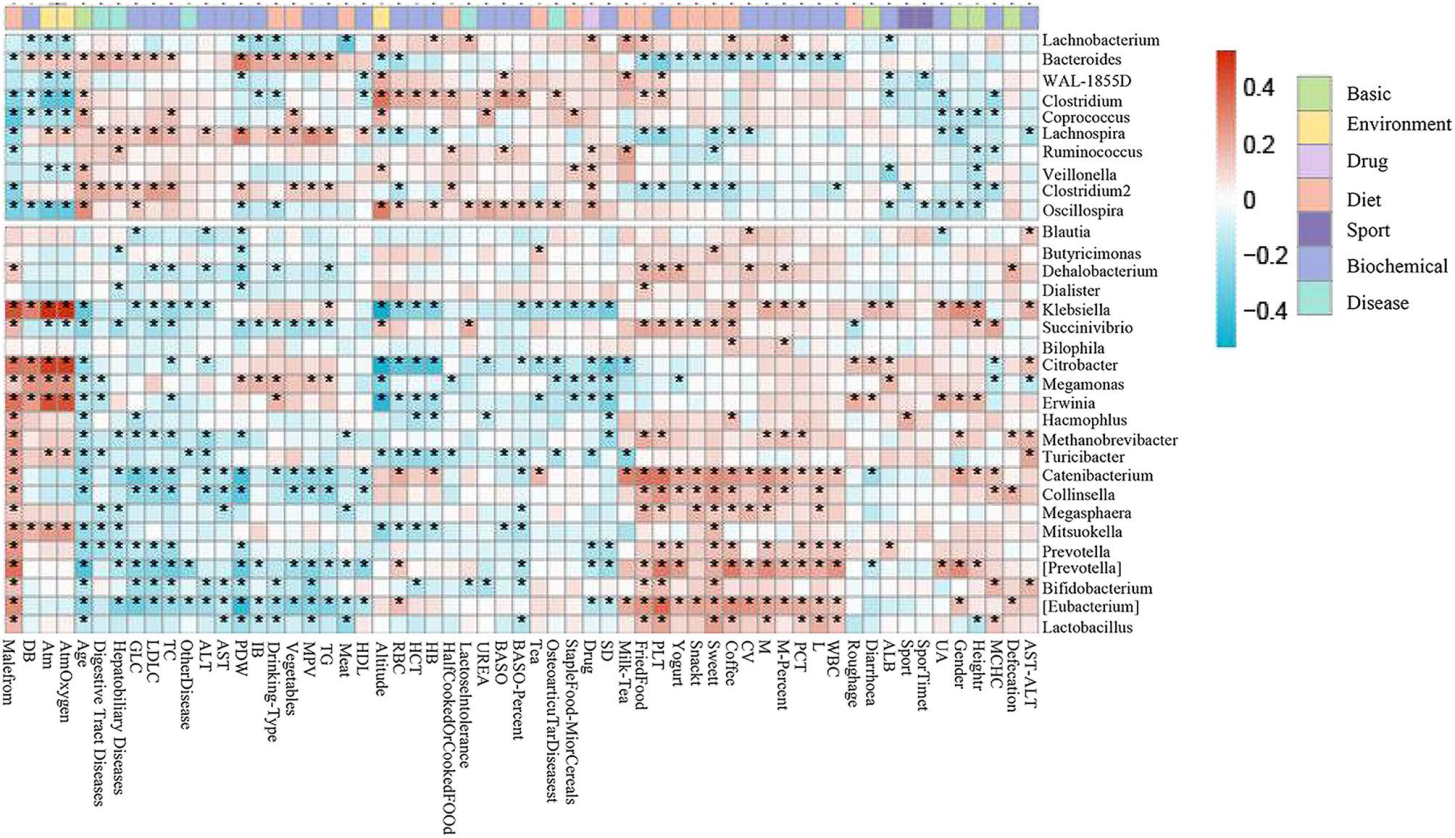
Correlation analysis of gut microbiota with external factors in Tibetans with high Incidence. Association diagram of intestinal microbes with high incidence and external factors in Tibetan population: The horizontal axis represents each influencing factor and clinical indicators, and the vertical axis represents the differential microbes. “*” in the figure represents that there is a significant correlation between the corresponding species-factors (*P* < 0.05). The red and green blocks in the figure represent the magnitude of correlation R, ranging from − 1 to 1, and the blue (negative) represents the negative correlation between the two. (red) positive value indicates that there is a positive correlation between the two. The larger the absolute value is, the greater the correlation is and the redder the color is, indicating that this factor shows an upward trend with the increase of the abundance of bacteria. The bluer the color is, the opposite is true.

**Figure 4:**
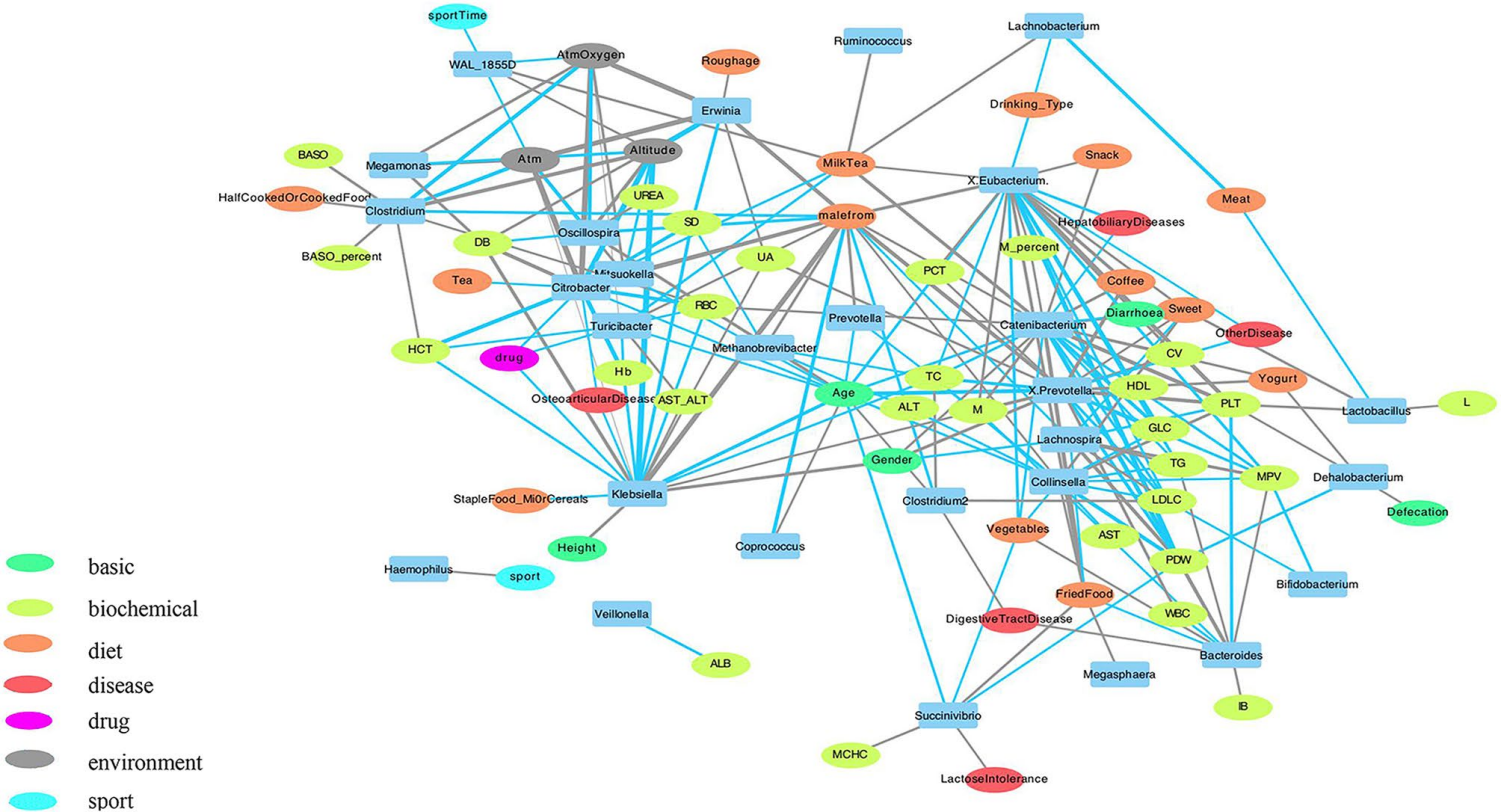
Correlations between different species and environmental factors and clinical indicators. Network diagram of intestinal microbes and significantly related factors in Tibetan population with high incidence. The blue line represents the negative correlation, the gray line represents the positive correlation; Line thickness on behalf of the correlation between size (here select|r|> 0.2, *p* < 0.05); Dots of different colors represent different factor types, and blue represents species.

In order to further explore the influencing factors of intestinal microbes in Tibetan population, we measured the relationship between the influencing factors and the characteristic changes of intestinal microbes in Tibetan population from the perspectives of diet index, disease index, medication index and environmental index. Based on the relative abundance table and factor information table of different species, Spearman correlation analysis in R software (Version 3.3.3) was used to measure the potential association between intestinal microbes and changes in clinical indicators. We established the association network between external factors-microbiome and clinical indicators (Fig. 3) to reveal the association between microbiome and the occurrence and development of high-incidence diseases as well as the potential mechanism. As can be seen from the figure, with the increase of altitude, the relative abundance of *Clostfridium* increases as atmospheric pressure and oxygen partial pressure decrease. Klebsiella was on the contrary. With the increase of atmospheric pressure and oxygen partial pressure, the relative abundance of altitude decreased and increased. Citrobacter was positively correlated with atmospheric pressure and partial oxygen pressure, but negatively correlated with altitude, RBC count, hematocrit, and hemoglobin content, suggesting that the relative abundance of Citrobacter increased with the increase of atmospheric pressure and partial oxygen pressure and the decrease of altitude, which might lead to changes in RBC count, hematocrit, and hemoglobin content. It can also be seen from the figure that coffee, fried food, meat, and other dietary factors are also significantly correlated with bacteria. In order to more intuitively see the correlation between different species and environmental factors and clinical indicators, we constructed a network diagram among the three (Fig. 4).

### Data search and navigation

**Figure 5:**
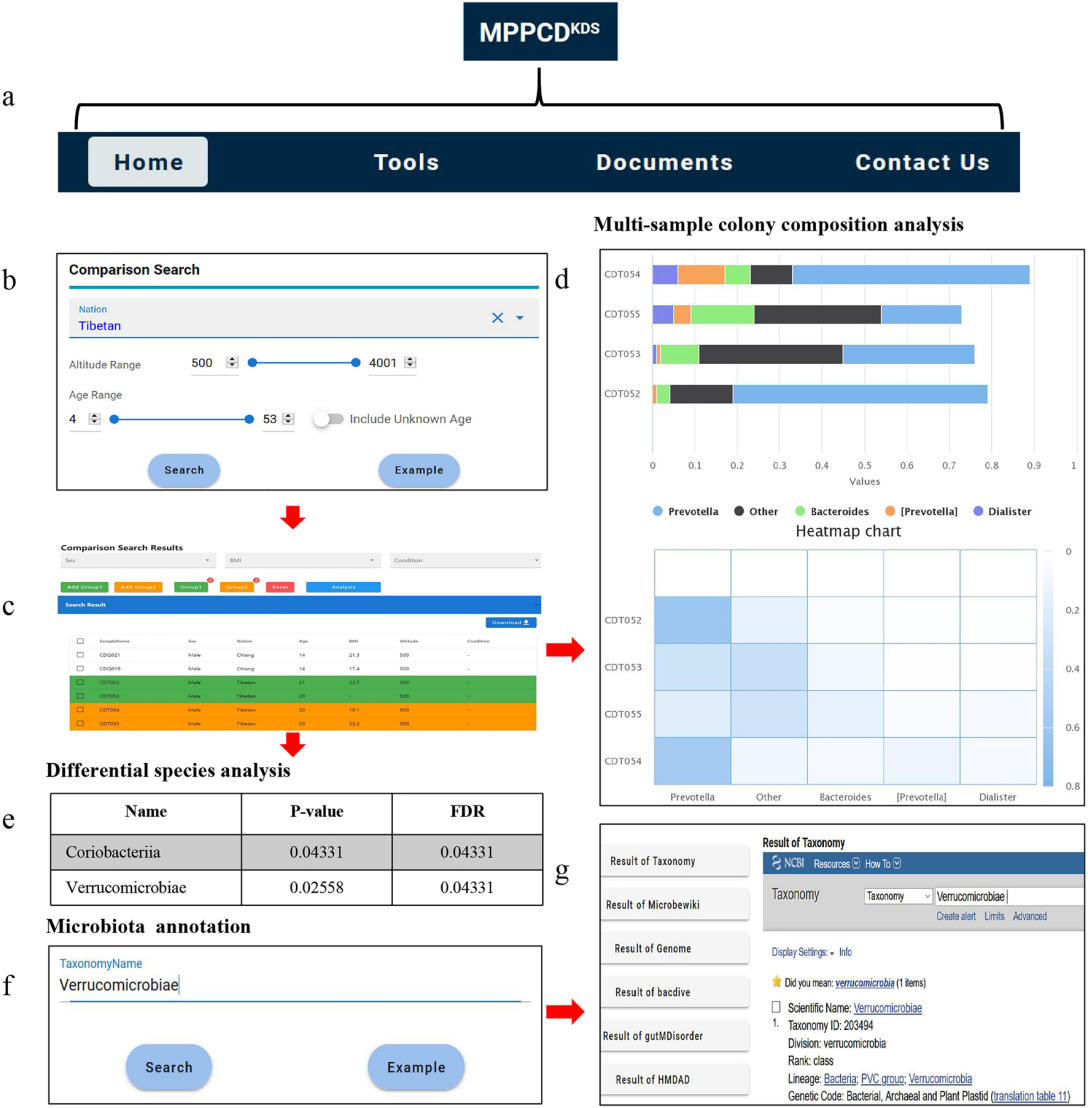
Screenshots and search functions of MPPCD. (**a**) The main modules of the website interface in MPPCD. (**b**) The interface for comparison search. (**c**) The results of the comparison search. (**d**) The relative abundance ratio of each sample is shown by a bar chart (upper) and heatmap (below). (**e**) Differential analysis of the abundance of each gut microbe among different groups. (**f**) Interface of microbiota annotation. (**g**) Annotation results of the different aspects in different databases and banks.

MPPCD (website: http://mppmcdb.cloudna.cn/) provides users with a concise search engine and a user-friendly interface to access, browse and retrieve different data types and analysis results. The website interface is comprised of four sections: “Home”, “Tools”, “Documents”, and “Contact us” (Fig. 5a).

On the “Home” page, a brief introduction of MPPCD, search functionality, users’ scopes, a statistical summary of all samples, database update history, etc. were provided. The search functionality mainly had two search modules, including “comparison search” and “microbiota search”. The comparison search functionality is designed to provide the ability to search samples by particular characteristics, including sex, ethnicity, age, living altitude, BMI and health conditions (Fig. 5b,c). Then, a list of samples within the database that fulfilled the search criteria was presented (Fig. 5c). This database mainly allowed users to select two groups of samples and compare the gut microbe components, abundance, and differences in each group (Fig. 5c). If desired, it is also possible to use this functionality to search just a single group and obtain its associated data, such as genus abundance. Importantly, they were chosen without particular order or with cross selecting. Meanwhile, the number of selected samples was also presented in both Group 1 and Group 2 (Fig. 5c). Clicking the “analysis” button would show the following results: (1) the gut microbial composition and relative abundance value customized by one or two groups in the phylogenetic level of phylum, class, order, family and genus, which is displayed for browsing with bar charts (Fig. 5d). Placing the cursor over the bars in the graph will provide detailed information about the search results. Meanwhile, the shown number of gut microbes could be user-defined, and sometimes, the actual number of gut microbes may be less than the required number. (2) The relative enrichment of each microbe in each sample was displayed and viewed using a heatmap at any phylogenetic level (Fig. 5d). (3) The difference analysis of each microbe abundance among Group 1 and Group 2 was performed using the *P* value and FDR, which contributed to determining the association between the gut microbes and related factors (Fig. 5e). In the comparison search, living altitude also introduced MPPCD and was regarded as a related factor, which was distinguished from other databases of human gut microbiota, such as HPMCD^19^, gcMeta^16^, and MANTA^17^. In addition to the “comparison search” module, we further provided the microbiota search functionality. The gut microbe’s name can be input in the text field where we provided a fuzzy matching function, such as *Verrucomicrobiae* (Fig. 5f). Clicking the “Search” button, the MPPCD would present the detailed microbe annotation with a series of related databases and banks, including Taxonomy annotation (https://www.ncbi.nlm.nih.gov/taxonomy the Microbewiki database), (https://microbewiki.kenyon.edu/index.php), Genome annotation (https://www.ncbi.nlm.nih.gov/genome/), the Bacdive database^34–36^, the gutMDisorder database^37^, and HMDAD bank (http://www.cuilab.cn/hmdad) (Fig. 5g).

**Figure 6:**
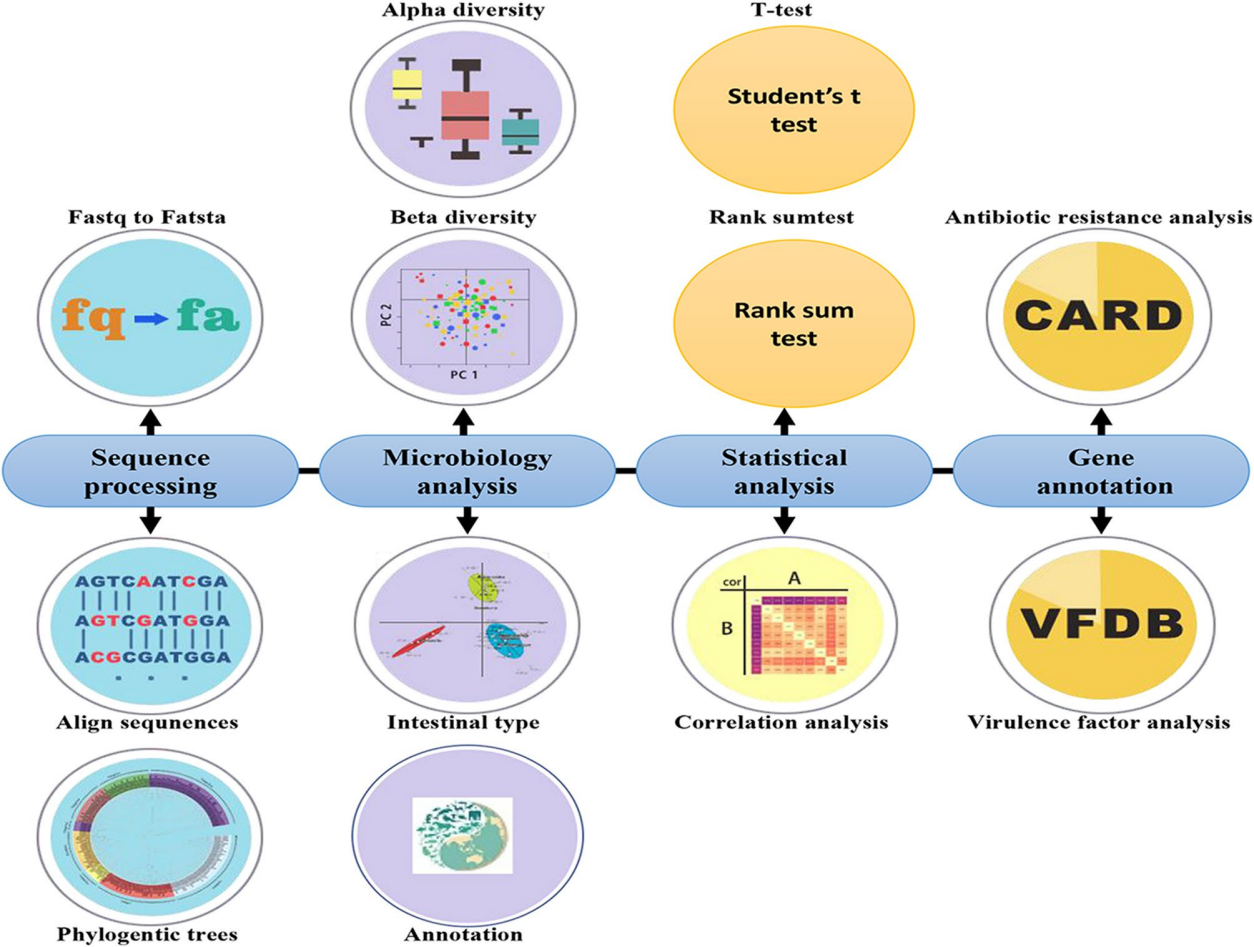
Functional descriptions of the Tool module. The module contains four sections: sequencing processing, microbiology analysis, statistical analysis and gene annotation. The corresponding tools were also presented.

On the “Tools” page, to better understand and analyze human intestinal microbes, MPPCD presents many analysis tools that are made up of four sections, including sequencing processing, microbiology analysis, statistical analysis and gene annotation (Fig. 6 and Table 1). All the tools can be used by clicking the icons (Fig. 6). Table 1 presents the application scenarios for all human gut microbes.

**Table 1:** The analysis tools of human intestinal microbes.

Name	Analysis tools	Function or application
Sequence processing	Fastq to fasta	To convert fastq file to fasta file with program language
	Align sequences	Multiple sequence alignment by Muscle software
	Phylogentic trees	To build the evolutionary tree with PhyML software that approved many files format
Microbiology analysis	Alpha diversity	To analyze how many different sequences (microbes) are in a single sample
	Beta diversity	To research differential analysis among different groups with PCoA measures
	Intestinal type	The samples are divided into different types by clustering algorithm
	Annotation	To analysis the composition of gut microbes in every samples
Statistical analysis	T-test	Differential analysis among different groups
	Rank sum test	Differential analysis among different groups
	Correlation analysis	To research the relation for many factors
Gene annotation	Antibiotic resistance analysis	To research the drug resistance analysis for gut microbes
	Virulence factor analysis	Virulence factor analysis for gut microbes

## Discussion

In this study, we succeeded in constructing an MPPCD database of intestinal microbes of minority people in Sichuan Province, China. It contained 1251 self-produced data instead of public database sources. Compared to other gut-related databases, MPPCD has many advantages.

One feature is the complete related factors with gut microbes. The gut microbiota community structures are affected by factors such as age and sex. At present, there are some human microbe-disease databases. HMDAD is a resource that collects and curates human microbe-disease association data from microbiota studies and contains these related factors^18^. MicroPhenoDB is regarded as the first database platform to detail the relationships between pathogenic microbes, core genes, and disease phenotypes in different body sites, which lack host lifestyle, age, and other factors^38^. HPMCD provides a detailed analysis of human microbial communities and supports research from basic microbiology and immunology for therapeutic development in human health and disease^19^. Not only do these related factors affect gut microbes, but it also contains ethnicity factors that were simply divided into European and Han Chinese. A larger number of studies have shown significant variations in microbiome composition in healthy individuals from different race and ethnicity categories^10,39–43^. In this MPPCD, these factors were included in MPPCD to clearly determine the association between microbes and diseases. Furthermore, we focused on specific regions, detailed race groups, and similar lifestyle and living environments. These factors contributed to exploring the relationship between microbes and diseases.

The second feature was altitude first imported into the MPPCD. Before, a string of studies had demonstrated that exposure to the hypoxic environment at high altitude played an important role in altering the gut microbial composition and its activities, which can cause gastrointestinal symptoms^44–50^. Adak et al.^44^ demonstrated that the gut microbial composition is altered during acclimatization to hypobaric hypoxia; the total aerobes decreased 50-fold, whereas total anaerobes increased 115-fold after 15 days of migration at high altitude. Kang et al.^47^ uncovered more *Enterobacteriales*, *Enterobacteriaceae*, *Gammaproteobacteria*, *Porphyromonadaceae* and *Escherichia Shigella*, and several other types of microbial species were found in the gut microbiota of the immigrant Han population living on the Tibetan Plateau than in that of the low-altitude Han population by sequencing the 16S rRNA V1–V3 regions. Lan et al.^48^ studied the correlations between the gut microbiota community structures of Tibetans and geography across six different locations ranging from 2800 to 4500 m and found significant differences in microbial diversity and richness, and *Faecalibacterium*, *Bacteroides* and *Bifidobacterium* were positively correlated with altitude, which suggested that gut microbiota may play important roles in regulating high altitude and geographical adaptations. Ma et al*.*^49^ found that bacterial taxa differed significantly among Tibetan sheep and sheep collected from high-altitude Tibetan Plateau (3000 m) and low altitudes (1800 m), respectively. Zhu et al*.*^50^ discovered that the gut microbiota of Han hypertensive patients from middle-altitude (2260 m) presented greater α—and β-diversities, a lower ratio of *Firmicutes/Bacteroidetes*, and a higher abundance of beneficial *Verrucomicrobia* and *Akkermansia* compared to Han hypertensive patients from low altitude (13 m). Among the healthy individuals, the gut microbiota also showed greater α-diversity and a lower ratio of Firmicutes/Bacteroidetes at middle altitudes than at low altitudes. These results suggest that altitude has a significant influence on gut microbiome compositions.

Another feature of this database is that we offer a set of data analysis and visualization platforms, including sequencing processing, microbiology analysis, statistical analysis, and gene annotation, which are made up of 13 analytical tools, such as correlation analysis and provide scientists with a robust site for powerful data analysis. Furthermore, the analytical tools are free, operate simply, and contain the samples.

In addition, several issues should be considered for MPPCD. First, the MPPCD is a relatively professional database and limits user groups, including microbiologists, immunologists and medical scientists. Second, the sample size is small. There are less than 1300 gut microbiota in MPPCD. As the diversity and size of samples increase to include diverse infections, disease conditions, ethnicity categories, and sample types in the future, the complexity and specificity of search functionality will be improved.

Third, the scope of the MPPCD database will also be widened from the current focus on the human gastrointestinal tract to encompass samples from other human body sites, including the lungs and bladder. In the next few years, the issues mentioned above are expected to be considered and solved in future versions.

In conclusion, MPPCD is the first association database between human intestinal microbes and diseases in China’s ethnic minorities and contains a series of search functionality and analysis tools. The search functionality mainly consists of “comparison search” and “microbiota annotation”. The analysis tools include four modules: sequencing processing, microbiology analysis, statistical analysis, and gene annotation. Importantly, the residential altitude was first regarded as a related factor introduced into the MPPCD database. Furthermore, we found differential gut microbes among customized groups at the phylum, class, order, family, and genus levels.

## Methods

### Ethics committee approval

All clinical and experimental steps were approved by the ethical committee of Chengdu Medical College (NO. 2017009). All experiments were performed in accordance with relevant named guidelines and regulations. All participants received written informed consent and signed informed consent.

### Subject selection and sampling

Human subjects included in the MPPCD database were acquired from rural and urban people of Sichuan Province, including those from Chengdu city, Hongyuan County, Barkam County, and Wenchuan County. We visited the relevant areas and completed data collection with the assistance of local health authorities^51^. All the individuals (natural crowds) resided in different regions of Sichuan Province at altitudes ranging from 500 to 4001 m. They belonged to different ethnicities, including Tibetan, Han, Chiang, Hui, Yi and Mongol Chinese. The subjects completed a questionnaire survey, including demographic information (age, sex, birthplace, place of residence, ethnicity, etc.), health status(digestive tract diseases, type 2 diabetes, mental health, genetic diseases, etc.), diet (staple food, dietary intake, drinking habit, consumption of coffee, tea, yogurt, etc.) and exercise(daily physical activity, exercise frequency, etc.). A total of 1251 fecal samples were collected for analysis. These samples are included in the database, where they raised from human faecal samples and contain sufficient metadata to determine if they originate from a healthy or diseased individual, which were immediately transported to the laboratory of Chengdu Medical College with an ice pack and then frozen at − 20 °C. Meanwhile, all clinical information was collected according to standard procedures, including age, sex, body mass index (BMI), ethnicity, living altitude, height, weight, blood pressure and physical condition. Fasting peripheral venous blood was collected by trained personnel at all collection points according to standardized procedures for routine blood tests (hemoglobin, red blood cell, white blood cell and platelet counts) and biochemical tests (liver and kidney function, blood glucose and blood lipid levels). During the period of data collection, check the data regularly and carry out quality control.

### DNA extraction and 16S ribosomal RNA sequencing

Microbe DNA of every sample was extracted at Novogene Bioinformatics Technology Co., Ltd. using a TianGen Kit (TIANGEN BIOTECH (BEUJING)Co., LTD) according to the manufacturer’s recommendations, and then DNA concentrations were quantified using a Qubit 2.0 Fluorometer (Invitrogen). The V3-V4 hypervariable regions of the 16S rRNA of bacteria and archaea were amplified using the common primers 341F (5’- ACTCCTACGGGA GGCAGCAG -3’) and 806R (5’- GGACTACHVGGGTWTCTAAT -3’). The purified PCR products were constructed with a short fragment library with a 2 × 250 paired-end (PE) configuration and sequenced using the Illumina HiSeq platform. The required sequencing results were performed by bioinformatic and statistical analysis. The dataset supporting the results of this article has been deposited in the EMBL European Nucleotide Archive (ENA) under BioProject accession code PRJEB44310.

The raw reads were filtered to remove low-quality and polyclonal sequences using QIIME (Version 1.9.1) software^52^. The filtered data were further compared with the Gold database, and the chimaera reads were detected by using the Uchime algorithm in Usearch software (Version 8.1.1861)^53^. The resulting reads for each sample were clustered into operational taxonomic units (OTUs) at the level of 97% similarity using the Uclust algorithm in QIIME^52^. Representative sequences for each OTU were selected, and the taxonomic information was annotated using the Greengenes database (Version 13.8)^54^. In addition, we selected 5 diseases with high prevalence in Tibetan population, including rheumatoid arthritis, hyperlipidemia, hypertension, chronic gastritis, and hyperuricemia. According to the available data of each sample, the difference of OTU abundance between each disease group and the normal group was analyzed. Finally, we also analyzed the influence of external factors on intestinal microbes of Tibetan population. Based on the relative abundance tables and factor information tables of different species, Spearman correlation analysis in R software (Version 3.3.3) was used to measure the potential association between intestinal microbes and changes in clinical indicators.

### Statistical analysis

Based on the results of taxonomic analysis, the microbe types and relative abundance ratio of each sample are shown in the form of bar charts and heatmaps at the phylum, class, order, family, and genus levels using R programs. We also performed differential analysis of the microbe abundance among different groups with LEfSe software^55^. The *P*-value was determined by the Wilcox rank-sum test, indicating the differential analysis was performed by both the stats package of R language and the script package of python language, and then, it was adjusted by the false discovery rate (FDR) measures.

### Database schema and implementation

The underlying MPPCD Web server was based on the MongoDB database and Galaxy platform to manage and schedule the analysis tools. Analysis applications were implemented with Python, Bash and R languages. The webpage and interactions of MPPCD were accomplished by the JavaScript and Cascading Style Sheets (CSS) languages.

### Supplementary Information

Supplementary Information.

## Data Availability

The dataset supporting the results of this article has been deposited in the EMBL European Nucleotide Archive (ENA) under BioProject accession code: PRJEB44310. Any remaining data are available from the corresponding author upon reasonable request.
