# Supplementary material for: A display and analysis platform for gut microbiomes of minority people and phenotypic data in China
Source: Sci Rep. 2023 Aug 30;13:14247. doi: 10.1038/s41598-023-36754-5 (PMC10469205; doi:10.1038/s41598-023-36754-5)
Supplement: Supplementary file 1 — Supplementary Information. [file 41598_2023_36754_MOESM1_ESM.pdf]

Supplementary Material

Supplementary Figures

Fig. S1: Heat map of different species relative abundance of inter-groups.

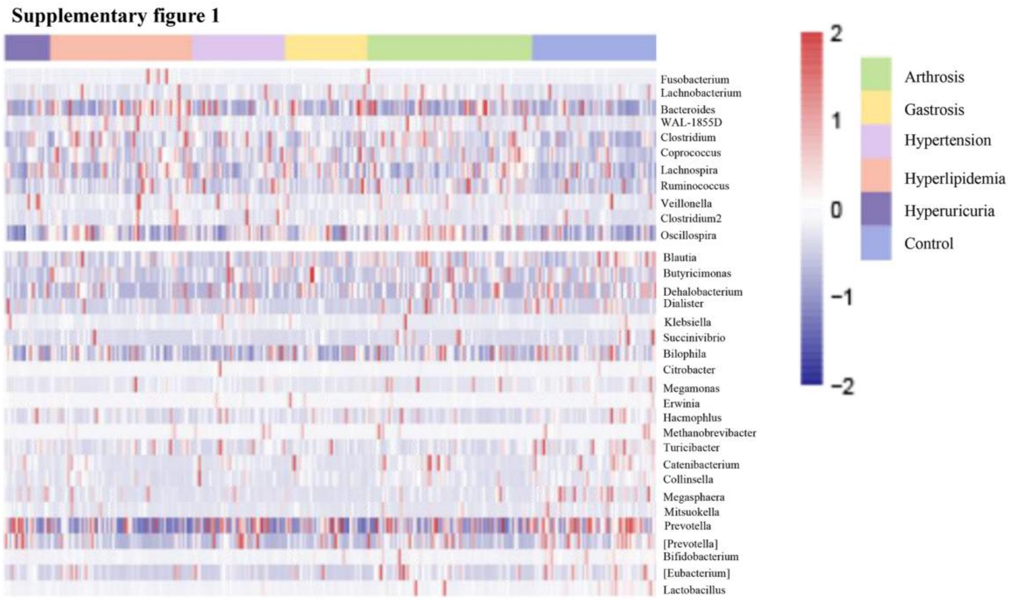

The transverse axis represents a sample of a single disease, and different colors represent different types of diseases, and the vertical axis represents the difference species. The different colors of the heat map indicate the abundance of the species in the corresponding sample(Red means higher abundance, blue means lower). On the right strip, the relative abundance value of the species represents the new value obtained after the homogenization is processed.This heat map was generated by corresponding author Li Jun using R language pheatmap package（version number:R-4.0.2,URL link:https://cran.r-project.org/;version number:RStudio Windows 10/11,URL link:https://posit.co/）, data from the MPPCD database.
